# Prescription Department

**Published:** 1892-06

**Authors:** 


CARBUNCLES AND BOILS.—By *F. A. Alford, M. D.*, Ferndale, Cal.

R. Hydrarg. oxidi rubri, dr. j.
Acidi carbolici, gr. x.
Gum camphor, gr. xx.
Menthol, gr. xv.
Morph. sul. gr. v.
Cocainæ hydrochlor. gr. v.
Lanolin, } aa parts æquales ad
Vaselin, } unciam j. m.

Sig. Triturate carbolic acid, camphor and menthol until liquified; then add the morph. sul. and cocaine, and lastly the mercury. Apply constantly and very freely, with no poultices.

COLD IN THE HEAD.—

A good treatment for this, if the sufferer must be out and about his work is to use a snuff composed as follows:

R. Menthol, 3 gr.
Powd boracic acid, dr. j.
Subnitrate of bismuth, dr. 1 1-2.
Powd. benzion, dr. 1 1-2. M.

Sig. A good-sized pinch of this may be snuffed up five or six times a day; and it will not only relieve the stuffiness and the other disturbing symptoms, but promote a rapid cure.

CATARRHAL AFFECTIONS.—

An excellent cleansing and disinfecting solution for free use in the nasal cavities, by means of the spray apparatus, douche or syringe, is prepared as follows:

R. Acidi boracici, dr. i.
Sodii borat. dr. i.
Sodii chloridi, dr. ss.
Listerine, oz. ii.
Aquæ puræ, oz. vi. M.

CHRONIC GASTRITIS.—

Stearns' Cascara Aromatic, 1 fl. oz.
Tr. Cardamons comp., 1 1-2 fl. dr.
Fl. ext. wood betony, 1 fl. oz.
Simple syrup, q. s. ad, 4 fl. oz.

Mix. Sig. Teaspoonful three times a day before meals.

OTORRHŒA.—

R. Eucalyptol 1-2 dr.

Sodæ bicarbonas, 15 gr.

Sodæ biborat. 15 gr.

Glycerine, 1 dr.

Aquæ ad. q. s., 2 oz.

M. S. Shake, and use as a lotion for the ear.

CHRONIC RHEUMATISM.—
The following has been recommended:

R. Acidi arsenioci, gr. j.
Pulv. guaiaci, dr. j.
Pulv. capsici, gr. x.
Pil. aloes et asafœt, dr. j.

M. et ft. pil. No. xl. S. One pill three times a day.

FOR HABITUAL CONSTIPATION.—
R. Extract. cascaræ sagrad. fl. dr. j
Tinct. nucis vomicæ, m. x.
Tincture belladonnæ, m. v.
Aquæ, oz. j.

M. Ft. haustus. S. To be taken twice a day.—DR. JAMES D. STAPLE, in the *Hospital Gazette.*

FOR THE DRY, RINGING COUGH OF INFLUENZA the following is a good combination:
R. Syrupi picis, oz. jss.
Spts. ammoniæ arom, oz. ss.
Syrupi pruni virg. oz. jss.
Potassii iodidi, dr. ss.

M. S. Teaspoonful 2 to 4 hours apart.—*North Carolina Medical Journal.*

BURNS.—
The following is an excellent dressing for burns:
R. Campho-phenique, oz. j.
Lanolin,
Ung. aquæ rosæ, aa oz. j.

M. Sig. Apply two or three times a day.—*Weekly Medical Review*, Mar. 12th, 1892.

MORNING LAXATIVE.—By *Prof. Henry F. Lyster*, Detroit, Mich.
R. Epsom salts, oz. jss.
Zingiber, pulv. dr. ij. M.

Sig. Two-thirds teaspoonful to wine-glass of water, before breakfast.

DYSPEPTIC VERTIGO.—
Dr. W. K. Harris, of Mulvane, Kan.' uses for dyspeptic vertigo:
R. Acidi nitrici, 60 drops.
Acidi muriatici, 100 drops.
Aquæ camphoræ, 8 oz.

M. Sig. Shake. Take one tablespoonful in one-half cup of water thirty minutes before each meal.

HYPODERMIC ERGOTIN INJECTIONS.—By *Prof. Biedert.*
R. Ergotin, 1.0.
Aq. dest., 5.0.
Acid carbol. 0.01. M.

This solution is totally free from irritating qualities and furthermore keeps for a long time.

